# CeO_2_ Nanomaterials from Diesel Engine Exhaust Induce DNA Damage and Oxidative Stress in Human and Rat Sperm In Vitro

**DOI:** 10.3390/nano10122327

**Published:** 2020-11-24

**Authors:** Martina Cotena, Mélanie Auffan, Stéphane Robert, Virginie Tassistro, Noémie Resseguier, Jérôme Rose, Jeanne Perrin

**Affiliations:** 1 IMBE, CNRS, IRD, Avignon Université, Aix Marseille University, 13005 Marseille, France; martina.cotena@univ-amu.fr (M.C.); virginie.tassistro@univ-amu.fr (V.T.); 2 CEREGE, Europole Arbois, BP 80, CNRS, IRD, INRAE, Coll France, Aix Marseille University, 13545 Aix en Provence, France; auffan@cerege.fr (M.A.); rose@cerege.fr (J.R.); 3 Civil and Environmental Engineering, Duke University, Durham, NC 27708, USA; 4 AMUTICYT Core Facility, UMR1263, INSERM, INRAE, Aix Marseille University, 13005 Marseille, France; stephane.robert@univ-amu.fr; 5 Department of Biostatistics and Public Health, La Timone Hospital, 13005 Marseille, France; noemie.resseguier@univ-amu.fr; 6 Laboratory of Reproduction Biology-CECOS, Department of Gynecology, Obstetrics and Reproductive Medicine, AP-HM La Conception, Pôle Femmes Parents Enfants, 13005 Marseille, France

**Keywords:** nanoparticles, DNA damage, Oxidative stress, reproductive toxicity, combustion, ageing, NMs life cycle

## Abstract

Cerium dioxide nanomaterials (CeO_2_ NMs) are widely used in nano-based diesel additives to decrease the emission of toxic compounds, but they have been shown to increase the emission of ultrafine particles as well as the amount of released Ce. The Organization for Economic Cooperation and Development included CeO_2_ NMs in the priority list of nanomaterials that require urgent evaluation, and the potential hazard of aged CeO_2_ NM exposure remains unexplored. Herein, human and rat sperm cells were exposed in vitro to a CeO_2_ NM-based diesel additive (called Envirox^TM^), burned at 850 °C to mimic its release after combustion in a diesel engine. We demonstrated significant DNA damage after in vitro exposure to the lowest tested concentration (1 µg·L^−1^) using the alkaline comet assay (ACA). We also showed a significant increase in oxidative stress in human sperm after in vitro exposure to 1 µg·L^−1^ aged CeO_2_ NMs evaluated by the H_2_DCF-DA probe. Electron microscopy showed no internalization of aged CeO_2_ NMs in human sperm but an affinity for the head plasma membrane. The results obtained in this study provide some insight on the complex cellular mechanisms by which aged CeO_2_ NMs could exert in vitro biological effects on human spermatozoa and generate ROS.

## 1. Introduction

Nanoparticles (NPs) and nanomaterials (NMs), particularly metal oxide NMs, are increasingly used in many fields of everyday life, e.g., food packaging, cosmetics, textiles, electronics, and even biomedicine. Extensive usage of NMs in various areas has raised human health concerns, mostly in terms of occupational exposure [1,2]. Considering the life cycle of nano-enabled products (from the production and formulation stage to their usage and end of life), the occupational exposure of workers to NMs [3] has been more studied than others viz., consumer and environmental exposures. The reason is the presence of non-nano-specific regulations, which put worker protection at the forefront with efficient individual protection equipment [4]. Consequently, major knowledge gaps remain in the risk assessment of NMs, especially in the post-production stages of their life cycle [5], e.g., people/consumers are directly or indirectly exposed during use or during waste treatment.

Among all, CeO_2_ NMs have been increasingly used in Europe and elsewhere as fuel-borne catalysts in diesel engines [6,7] as the Envirox^TM^ from Energenics Europe Ltd. The addition of CeO_2_ NMs in diesel has been reported to increase the fuel combustion efficiency [8] and decrease the emission of CO_2_, CO, the total particulate mass, formaldehyde, acetaldehyde, acrolein, and several polycyclic aromatic hydrocarbons [9] during combustion. However, it has also been shown to increase the emission of ultrafine particles, NOx, and benzo[a]pyrene as well as the amount of Ce released in natural environments (air, water, soil) near roads [10,11]. In the UK, near an urban road where Envirox^TM^ is known to be used, an increase in ambient Ce-based NMs was observed with a concentration of ∼0.3 ng·m^−3^ and aerodynamic diameters peaking at 150 nm [12]. To date, there are large uncertainties regarding the acceptable level of Ce-based NMs in the atmosphere because most toxicity studies have been done with pristine CeO_2_ NMs that are not representative of the emission in diesel exhaust. Indeed, once released in the atmosphere after combustion in a diesel engine, Ce is in the form of CeO_2_ NMs with different physical-chemical properties compared to pristine CeO_2_ NMs (in terms of size, surface properties, mineralogy, aggregation state, solubility) [4], which can affect their fate, bioavailability [13,14] and potential toxicity [2,15]. Therefore, investigating the potential adverse effects of such combusted CeO_2_ NMs on human health represents an important step in the safety assessment required from Registration, Evaluation, Authorisation and Restriction of Chemicals (REACH) and from the Organization for Economic Cooperation and Development (OECD’s guidelines 2011) [16,17].

Atmospheric pollution is known to affect numerous physiological functions, including human reproduction and fertility, and it is reported to be related to lower semen quality [17,18,19,20,21,22], reduced fertility and spontaneous fertility rate [18,19,20], and reduced success rates of in vitro fertilisation (IVF) in humans [21,22]. Regarding the reprotoxicity of NMs, controversial reports have been published in recent years with particular attention to the effects on male gametes [23]. Few in vivo and in vitro studies have estimated the potential harmful impacts of CeO_2_ NMs on reproductive organs and germ cells. Pristine CeO_2_ NMs (5–40 nm) have been shown to cross the blood–testis barrier and accumulate in the testis (<0.2% of the inhaled dose) of rats following 28 days of inhalation in vivo (total estimated inhaled dose 0.83–4.24 mg/rat CeO_2_ NMs) [24]. Accumulation of Ce in the testes of mice was also observed after 32 days of in vivo oral administration of pristine CeO_2_ NMs (27.62 ± 3.01 nm) at the highest doses of 20 or 40 mg/kg body weight [25]. This accumulation caused a decrease in daily sperm production, lower motility, and sperm DNA damage [25]. Our team recently demonstrated the in vitro genotoxicity of pristine CeO_2_ NMs (7 nm) on human sperm cells and mouse gametes after exposure to 10 µg·L^−1^ [26,27]. Significant impairments in fertilisation rates were observed in mice [26], and a significant increase in DNA damage in human sperm [27]. The mechanisms of DNA damage were indirectly attributed to oxidative stress via the adjunction of an antioxidant (L-ergothineine) in the exposure medium [27].

Most of the previous studies were conducted with pristine CeO_2_ NMs (i.e., at the production stage of the life cycle), which does not reflect a realistic exposure route of men and women to Ce-based NMs that are likely released in diesel exhaust. However, the WHO’s International Agency for Research on Cancer classified diesel engine exhaust as carcinogenic to humans [28], and the OECD classified CeO_2_ NMs as part of a priority list of NMs whose potential toxicity require urgent evaluation [16]. Hence, the potential reproductive toxicity [29] of combusted CeO_2_ NMs must be evaluated.

This study was designed to evaluate the potential genotoxicity induced by in vitro exposure of human and rat sperm cells to low concentrations of combusted commercialised CeO_2_ NM-based diesel additives (Envirox^TM^). Combining biological assays with physico-chemical characterisation, we addressed two questions: (i) Do combusted CeO_2_ NMs from a diesel additive induce genotoxic effects towards human and rat sperm cells following in vitro exposure to low concentrations? (ii) Were the mechanisms of genotoxicity and the interactions with sperm cells different from pristine CeO_2_ NMs?

## 2. Material and Methods

### 2.1. Physical-Chemical Characterisation 

#### 2.1.1. Ageing of the Diesel Fuel Additive

CeO_2_ NMs were recovered from Envirox^TM^, Energenics Europe Ltd., Oxfordshire, UK, which is a fuel-borne catalyst scientifically and commercially proven CeO_2_ NM-based diesel additive supplied by Energenics Europe Ltd. Envirox^TM^ was combusted following the protocol published in [4]. Briefly, we ultracentrifuged Envirox^TM^ suspensions at 396,750× *g* and 20 °C for 1 h and removed the supernatant to recover the pellet containing CeO_2_ NMs. The pellets were freeze-dried (Heto PowerDry LL3000, Thermo Fisher Scientific, Strasbourg, France) for 5 days. The dried samples were crushed, and a total amount of 1.2 g was introduced in a furnace at 850 °C (i.e., at the average combustion temperature of diesel engines) [30] for 20 min. A stock suspension of the combusted Envirox^TM^ (called aged CeO_2_ NMs) was prepared in Milli-Q water at 10.15 g·L^−1^ of CeO_2_. The combusted Envirox^TM^ contained CeO_2_ NMs with an average TEM size of 19 ± 10 nm, a larger polydispersity than the uncombusted ones, and a polyhedral shape [4]. We used X-ray diffraction (XRD) (X′Pert-Pro diffractometer, PANalytical) and transmission electron microscopy (TEM) (FEI 2Tecnaiï G2) to confirm the size, shape, and mineralogy of the produced NMs (see Supplementary Materials, Figure S1).

#### 2.1.2. Aged CeO_2_ NM Dissolution in FertiCult^®^ Medium

We assessed the dissolution of aged CeO_2_ NMs in FertiCult^®^ medium (JCD Laboratories, Lyon, France) for the in vitro culture of mammalian gametes by inductively coupled plasma mass spectrometry (ICP-MS) (NexION 300X, Perkin Elmer^®^). We incubated aged CeO_2_ NMs at room temperature (RT) in the culture medium for 2 and 5 h at three concentrations (10, 1000, and 100,000 µg·L^−1^). After incubation, the suspensions were ultra-filtered at 3 KDa (Amicon Ultra-15, Merck, France) at 3000× *g* for 1 h and by ICP-MS; triplicates were performed for each concentration.

### 2.2. Gamete Collection

*Rat sperm cell collection.* After sacrifice, we collected and cut the epididymis to enable the exit of sperm in HTF-BSA culture medium (Human Tubal Fluid, Millipore^®^, France, with 0.4% BSA: Bovine Serum Albumin, Sigma-Aldrich^®^, Lyon, France) for 1 h at 37 °C and CO_2_ 5%.

*Human sperm collection.* We used frozen human sperm from healthy fertile donors. After thawing, we aliquoted the preparation and centrifuged it for 10 min at 420 g. The supernatants were discarded, and the pellets were exposed to various exposure conditions.

### 2.3. Ethical Authorization

Ethical authorization for animal sampling of gametes was obtained from the National Ethics Committee on Animal Experimentation (2018061110211950-V2 #15447). We used Sprague Dawley rats, Oncins France Strain A (623OFA), which were purchased from Charles River Laboratories, France. Sexually mature male rats of 60 days old were housed with free access to food and water until sacrifice. 

Human sperm cells were purchased from GERMETHEQUE biobank, which obtained informed consent from each donor for inclusion of samples in the biobank and for their use in research experiments regarding human fertility in accordance with the 1975 Helsinki Declaration on human experimentation.

For oxidative stress analysis, the principle of Replacement of the 3R rule (reduction, refinement, and replacement) was applied and human sperm only was used.

### 2.4. Sperm Exposure

We exposed human and rat sperm to four different concentrations of aged CeO_2_ NMs (1, 10, 100, and 1000 µg·L^−1^) for 1 h at 37 °C and 5% CO_2_. FertiCult^®^ medium was used as a negative control and 110 µmol·L^−1^ H_2_O_2_ as a positive control. The H_2_O_2_ concentration was chosen based on previous studies [26,27,31]. At least three different experiments were performed. After exposure, we recovered all motile sperm cells by swim-up [32], and before comet assay analysis, we measured sperm viability by eosin-nigrosine staining according to the World Health Organisation (WHO, 1999, Appendix IV.2) technique (100 cells were evaluated per condition).

### 2.5. DNA Damage Evaluation by Comet Assay

We performed the alkaline comet assay according to the procedure described by Singh [33] and adapted by Baumgartner [34], which has already been described in previous publications by our team [26,27,31]. DNA damage was quantified by the percentage of DNA in the tail of 100 randomly selected sperm cells from each triplicate slide per condition (at least 300 raw values analysed per experiment, at least 900 in total per condition).

*Statistical Analysis.* The data presented the medians of % Tail DNA values, with 1st and 3rd quartiles. We performed a linear mixed model analysis with “condition” (exposure condition) as a fixed effect and “cells” (sperm cells) within the replicate slide as a random effect using linear mixed effects regression (lmer) function of R software, version 3.6.0 (R Foundation for Statistical Computing, Vienna, Austria) to compare DNA damage among the various conditions. Pairwise differences of least-squares mean for all conditions were post-hoc assessed. Statistical significance was set at *p* < 0.05.

### 2.6. Oxidative Stress Analysis on Human Sperm

We investigated the effect on human sperm of aged CeO_2_ NMs in vitro exposure on the generation of reactive oxygen species (ROS), at the exposure concentration that had induced the higher DNA damage, i.e., 1 µg·L^−1^. At least three different experiments were performed. We used a 2′,7′-dichlorodihydrofluorescein diacetate (H_2_DCF-DA) probe [35]. The exposure protocol was readapted from Aitken et al. (2013) and Gallo et al. (2018) [36,37]. After thawing and dilution in FertiCult^®^ culture medium, we centrifuged human sperm for 10 min at 420× *g*, discarded the supernatant and added 10 µmol·L^−1^ H_2_DCF-DA [36] at 37 °C and 5% CO_2_ for 45 min to enable internalization of the permeable probe. Then, the sperm cells were washed and exposed to 1 µg·L^−1^ CeO_2_ NMs for 1 h at 37 °C and 5% CO_2_. We used H_2_O_2_ 110 µmol·L^−1^ as a positive control and FertiCult^®^ medium as a negative control. After 1 h, we recovered the motile sperm by swim-up selection. For vitality staining, we exposed the sperm to 4′,6-diamidino-2-phenylindole (DAPI) at 0.5 µg·mL^−1^ immediately prior to analysis by flow cytometry (CytoFLEX, Beckman Coulter, IN, USA). The results are only based on a live sperm population and expressed as the percentage of DCF-positive cells (expressing the fluorescence) in 100,000 events per condition in each replicate experiment (300,000 live cells in total per condition).

### 2.7. Imaging of Human Sperm Cells after In Vitro Exposure

Human sperm were exposed in vitro to 1 and 100 µg·L^−1^ of aged CeO_2_ NMs and selected by swim-up after 1 h. Non-exposed sperm cells were used as the control. Recovered motile sperm cells were washed with 0.1 M phosphate buffer, then fixed with glutaraldehyde 2.5% in 0.1 M phosphate buffer during 30 min at RT, and finally rinsed 3 times with 0.1 phosphate buffer. Samples were post-fixed with 2% osmium tetroxyde in 0.1M phosphate buffer during 30 min and washed 3 times with 0.1 M phosphate buffer. Progressive dehydration with 50% to 100% Ethanol bath was performed before embedding in Epon 812 (epoxy resin) from 33% to 100% EPON. Ultrathin sections (60 nm) were obtained using Ultracut-E ultramicrotome (Reichert-Jung, Southbridge, MA, USA) and contrast was performed using Uranyl acetate 5% for 12 min and dried at room temperature. Pictures were obtained using a JEM 1400 transmission electron microscope (JEOL, Tokyo, Japan) at 80 kV with a Megaview III camera under iTEM Five software (SIS Imaging, Münster, Germany). This procedure was described in [27]. 

## 3. Results

### 3.1. No Detectable Dissolution of Aged CeO_2_ NMs in Abiotic Conditions

The dissolution (<3 kDa) of CeO_2_ NMs in abiotic FertiCult^®^ medium (pH 7.2–7.5) was studied after 2 and 5 h of incubation at 10, 1000, and 100,000 µg·L^−1^. Regardless of the concentrations tested, no dissolution of the CeO_2_ NMs could be measured by ICP-MS in abiotic conditions. Below 3 kDa, the concentrations of dissolved Ce species were under the limits of detection (i.e., <0.002 µg·L^−1^). 

### 3.2. Higher DNA Damage Detected at the Lowest Concentration Exposure

**Figure 1:**
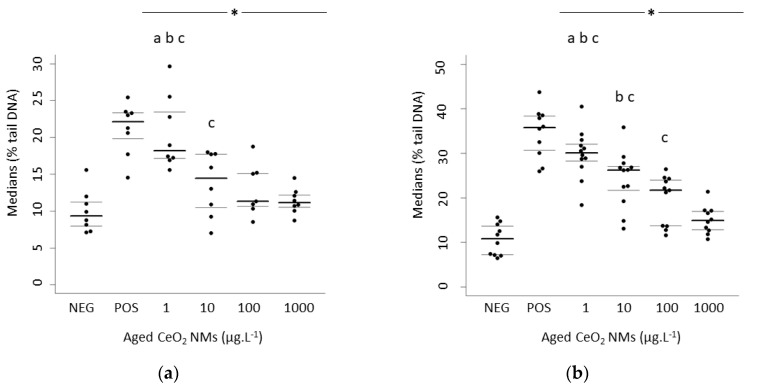
Evaluation of DNA damage using the Comet Assay following in vitro exposure of rat (**a**) and human sperm (**b**) to aged CeO_2_ NMs. Tested concentrations: 0, 1, 10, 100, and 1000 µg·L^−1^ of aged CeO_2_ NMs, POS (110 µM H_2_O_2_, positive control). *p* < 0.05, for differences compared versus *: negative control (NEG); a: vs. 10 µg·L^−1^ CeO_2_ NMs; b: vs. 100 µg·L^−1^ CeO_2_ NMs; c: vs. 1000 µg·L^−1^ CeO_2_ NMs. In each condition, the main central line corresponds to the median of the points and the two other lines correspond to 1st and 3rd quartiles.

Rat and human sperm cells were exposed in vitro for one hour to 0, 1, 10, 100, or 1000 µg·L^−1^ of aged CeO_2_ NMs. After exposure, all viability rates exceeded the normality threshold as stated by the WHO criteria [38]. The results are presented as the distribution of median values of % tail DNA with 1st and 3rd quartiles, obtained from 3 independent experiments. *p* < 0.05, for the differences compared versus *: negative control (NEG), a: vs. 10 µg·L^−1^ CeO_2_ NMs, b: vs. 100 µg·L^−1^ CeO_2_ NMs, c: vs. 1000 µg·L^−1^ CeO_2_ NMs (Figure 1a,b).

In rat- and human-exposed sperm cells, all exposure concentrations induced significantly higher DNA damage than the negative control (*p* < 0.05) (Figure 1a,b). Furthermore, a significant increase in DNA damage was observed at the lowest tested concentration (1 µg·L^−1^) (medians of % tail DNA in rats and humans of 18.20 and 30.10, respectively) compared to the exposure to 10, 100, and 1000 µg·L^−1^ aged CeO_2_ NMs; (*p* < 0.001). We also observed a significant difference between 10 and 1000 µg·L^−1^ aged CeO_2_ NMs in rat sperm (*p* < 0.05) and a significant difference among the three highest tested concentrations in human sperm (*p* < 0.05). The variability of biological data in the 3 independent experiments is presented in Table 1.

### 3.3. Oxidative Stress Detected in Human Sperm

**Figure 2:**
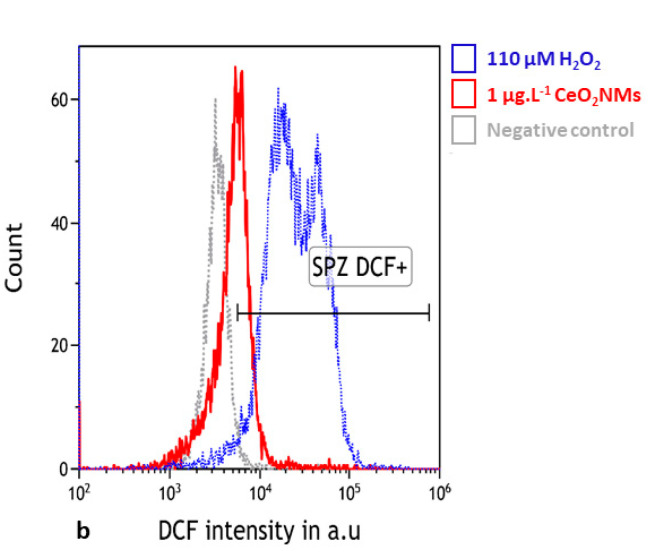
Detection of reactive oxygen species (ROS) using the H_2_DCF-DA probe. Human sperm cells were exposed in vitro for one hour to 1 µg·L^−1^ aged CeO_2_ NMs. Intracellular reactive oxygen species were evaluated by the dichlorodihydrofluorescein (DCF) intensity. The results are representative of 3 independent experiments. *p* < 0.05, for differences compared versus negative control. SPZ: spermatozoa. a.u: arbitrary unit.

After 1 h of in vitro exposure to 1 µg·L^−1^ aged CeO_2_ NMs, the percentage of DCF-positive live sperm (mean ± SEM= 13.1 ± 3.9) significantly increased compared to the negative control (5.21 ± 2.9) (*p* = 0.047) (Figure 2).

### 3.4. Aged CeO_2_ NMs Detected by TEM on the Plasma Membrane of Human Sperm

**Figure 3:**
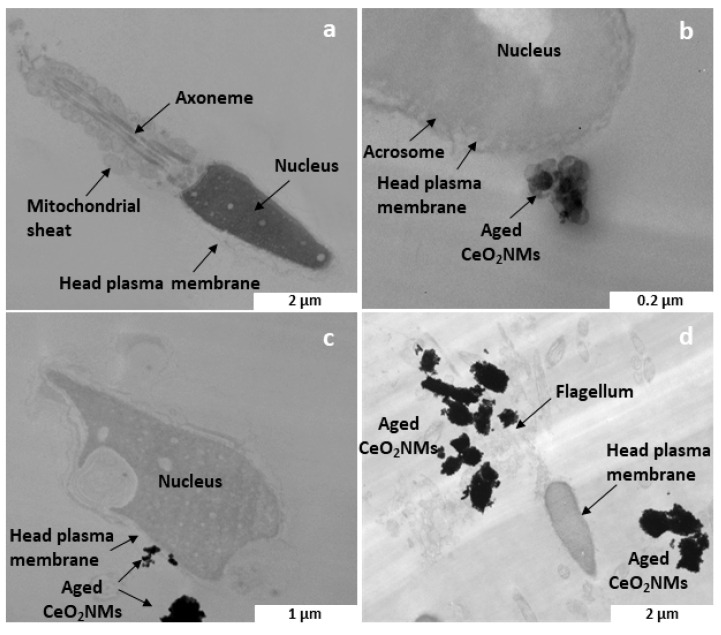
TEM analysis of aged CeO_2_ NMs in human sperm after in vitro exposure. (**a**) negative control; (**b**,**c**) exposed to 1 µg·L^−1^ CeO_2_ NMs; (**d**): exposed to 100 µg·L^−1^ CeO_2_ NMs. The white rectangles correspond to the scale bar.

In human sperm cells, TEM detected aged CeO_2_ NM aggregates near the sperm plasma membrane after the exposure to 1 µg·L^−1^ CeO_2_ NMs (Figure 3b,c). After the exposure to 100 µg·L^−1^, the NMs appeared even more aggregated and not in close interaction with the cells (Figure 3d). No CeO_2_ NM internalization was observed by TEM at any exposure concentration.

## 4. Discussion

### 4.1. Exposure to Low Doses of Aged CeO_2_ NMs Induced Higher DNA Damage 

One major drawback of the currently used studies and models to evaluate the toxicity of NMs for humans is the lack of relevance of the exposure scenario (i.e., relevant concentration of NMs, relevant speciation of the tested NMs) [39]. Indeed, most in vivo studies are based on theoretical data regarding the potential exposure of humans to CeO_2_ NMs. For example, Modrzynska (2018) exposed mice to a single dose of 162 μg of CeO_2_ or TiO_2_ NMs in 50 μL of 2% serum in nano-pure water [40]. This single dose corresponds to pulmonary deposition during 138-h working days at the Danish occupational exposure limit of 10 mg/m^3^ for TiO_2_ assuming 9% alveolar deposition [41,42]. Li (2016) exposed rats to CeO_2_ NMs in a nose-only exposure system for a single 4-h exposure (27–39.8 μg/m^3^). They used a scanning mobility particle sizer to estimate the average mass concentration and compared it with the physical sampling measurements based on the filter packs [43].

Here, we highlighted the importance of the dose and more particularly of the low dose. We expected a moderate release of CeO_2_ NMs in the air from a diesel engine and a spread into the gonads, as shown by Qin et al. (2019) and Préaubert et al. (2015) [25,26]. The lowest concentration of aged CeO_2_ NMs (1 µg·L^−1^) was found to induce the highest DNA damage in both human and rat sperm cells (Figure 1a,b). These results are consistent with our previous study, which showed inverse dose-response DNA damage after the in vitro exposure of human sperm to pristine CeO_2_ NMs [27]. Few hypotheses could be formulated to explain the highest genotoxicity at the lowest exposure concentration. One refers to different NMs/cells interactions because of dose-dependent aggregation states. Herein, it was not technically possible (below the detection limit) to measure the size of NMs aggregates at 1 µg·L^−1^ and 1000 µg·L^−1^ of CeO_2_ NMs in the FertiCult^®^ medium. However, the strong aggregation due to combustion already observed in reference [4] is also expected at the pH and ionic strength of extracellular media. Based on the dose-dependent probability of contact between NMs, smaller aggregates should be expected at the lowest concentration (1 µg·L^−1^ CeO_2_). In that case, smaller aggregates would be more prone to interact with the cells and enhance their biotransformation, biological, and toxicological effects [44]. It is known that nanoparticle genotoxicity and cytotoxicity are controversial, especially because these interactions are species-specific, often tissue-specific, and related to physico-chemical features of exposure medium [45,46]. Moreover, nanoparticles show a strong tendency to form agglomerates in solution due to their high surface area. It is a general opinion that the degree and type of agglomerates formed may influence the toxicity of NPs [47,48].

### 4.2. DNA Damage in Human Sperm Was Associated with Oxidative Stress 

A generally accepted paradigm is that the toxicity of NMs arises primarily because they can generate reactive oxygen species (ROS) and oxidative damage [44]. Oxidative stress is a major cause of DNA damage in mammalian spermatozoa [49], since it can affect the membrane integrity and motility [50,51], and it is also associated with failures of fertilisation, abnormal embryonic development, and premature pregnancy loss [52].

Understanding the mechanism of action of CeO_2_ NMs is a major challenge since they are known to exhibit pro- and antioxidant properties. For example, Das et al. (2007) [53] and Niu et al. (2007) [54] showed that CeO_2_ NMs (3–5 and 7 nm, respectively) could reduce oxidative stress as free radical scavengers, whereas Auffan et al. (2009) [55] highlighted the ability of CeO_2_ NMs (7 nm) to damage fibroblast DNA at very low doses (6–1.2 × 10^6^ µg·L^−1^). The determining factors are the stoichiometry, oxido-reduction state of Ce, pH of the medium, presence of H_2_O_2_, etc. [56], but it also depends on the cell types, organisms [57], size and speciation of the NMs [58].

Herein, we demonstrated in human sperm that 1 h of in vitro exposure to 1 µg·L^−1^ aged CeO_2_ NMs induced a significant increase in ROS generation. The H_2_DCF-DA probe becomes fluorescent on oxidation and is purported to directly monitor reactive oxygen and/or nitrogen species (ROS/RNS) [59]. ONOO- and the hydroxyl radical also directly oxidise this probe and significantly contribute to the positive signals observed in defective human spermatozoa [60,61]. Our results confirm previous studies, where we showed that DNA damage was induced in human sperm by exposure to 10 µg·L^−1^ of pristine CeO_2_ NMs was significantly decreased by the addition of an antioxidant (L-ergothioneine) in the exposure medium [27]. L-ergothioneine is known to scavenge hydroxyl radicals, hypochlorous acid, and peroxynitrite [62], which can directly oxidise the H_2_DCF-DA probes [60,61]. We hypothesize that oxidative stress can be one of the mechanisms responsible for the DNA damage detected in sperm cells. 

At 1 µg·L^−1^, aged CeO_2_ NMs were found in close contact with the plasma membrane of the head of the spermatozoa. This interaction with the sperm head associated with the highest sensitivity of the H_2_DCF-DA probe when oxidants are generated near the plasma membrane or in the cytoplasm [36] can help us elucidate the mechanisms of oxidative stress generated by aged CeO_2_ NMs. Indeed, the head of the spermatozoa expresses various ion channels (e.g., Ca^2+^ and K^+^) [63,64], which offer entry paths for metallic toxicants such as Zn^2+^ and Pb^2+^ into a mature spermatozoa [63,65,66]. More interestingly, some trivalent ions such as La^3+^ and Ce^3+^ act as T-type calcium channel antagonists and competitively bind and block the Ca^2+^ binding sites [67,68], which are required for the sperm head mannose receptors [65]. Then, mannose receptor expression is considered a biomarker for the effects of transition and heavy metals and organic toxicants on sperm fertility potential [66]. 

Here, we did not observe any abiotic dissolution of Ce in the Ferticult^®^ medium. However, reductive dissolution of nanocrystalline Ce(IV)O_2_ into Ce(III) is pH-dependent at pH < 7 [69,70,71] and it is possible that a release of Ce(III) with a pro-oxidant activity could occur in the vicinity of the cells due to the metabolic activity [69,70]. Indeed, in sperm cells, voltage-gated proton channels Hv1 (i.e., the main H(+) extrusion pathway) activated by alkaline extracellular environment induce an alkalinisation of intracellular pH necessary for the functional activation (capacitation) of sperm, and consequently a decrease in extracellular pH [72]. Such an extracellular pH decrease could locally enhance the dissolution of CeO_2_ NMs in the vicinity of the cells. More information on the biodistribution and biotransformation of CeO_2_ at the scale of the cell membrane is required to validate the hypothesis that the observed oxidative stress can be attributed to the possible biotransformation of CeO_2_ at the surface of the plasma membrane.

### 4.3. Lifecycle Stage-Dependent (Geno)Toxicity of CeO_2_ NMs

To date, few studies have explored the DNA damage and oxidative stress induced by pristine CeO_2_ NMs (mostly monodisperse and homogeneously coated), which are not representative of CeO_2_ NMs that are likely released during use. One strength of this study is the use of combusted Envirox^TM^ to mimic real exposure to CeO_2_ NMs, which are potentially released from diesel vehicles equipped with an active nano-catalyst for soot abatement. With an over-combustion in a single-cylinder engine with mechanical fuel injection [13] or in an oven at a temperature close to that of diesel engines [4], the physico-chemical properties of CeO_2_ NMs evolve compared to those initially incorporated in the diesel additive: the size of the CeO_2_ crystallites significantly increased (i.e., the decrease in specific surface area from 113 ± 17 to 63 ± 35 m^2^·g^−1^) without detectable Ce(III) in the structure, and no organic compounds remained at the surface. This transformation upon combustion highlights that at each stage of the nano-enabled product life cycle (formulation, usage/combustion, and end of life), the mechanisms and kinetics of interactions between the released CeO_2_ NMs and the aqueous media (e.g., water, soil, biological media) and exposed organisms will differ [5,73]. Hence, the life cycle stages of nano-enabled products when studying their toxicity is critical [62,74]. This is particularly the case when studying their genotoxicology [75] towards gametes since fertility may be altered, which subsequently affects the reproduction rate and health of the offspring [26,76].

Based on previously published data and the current study, we compared the impacts towards gametes of pristine CeO_2_ NMs (production stage of the life cycle) and combusted diesel additives (usage stage of the lifecycle). At both lifecycle stages, a higher genotoxicity attributed to oxidative stress was observed at the lowest doses towards human sperm cells [26,27]. For pristine and combusted NMs, an inverse dose–effect relationship was attributed to different aggregation states (size and density). At low doses, the probability of contact between two particles (homo-aggregation) decreases, and CeO_2_ NMs are likely more dispersed, which increases the contact surface between NMs and cells [27]. However, for pristine NMs only, the significant genotoxicity at low doses was also attributed to their chemical instability because of their larger specific surface area and an organic coating at the surface [4].

## 5. Conclusions

We demonstrated for the first time the DNA damage induced in human and rat sperm cells after exposure to low concentrations of CeO_2_ NMs, which are similar to the ones released by combustion in a diesel engine. In vitro exposure to the lowest concentration of combusted CeO_2_ NM induced oxidative stress in human sperm cells after the interaction with the plasma membrane. While important from a mechanistic standpoint, this study remains limited by its in vitro nature. However, in vitro exposure is a relevant design since spermatozoa can encounter nanoparticles in the male gonads or female genital tract. In vivo studies after a low-dose exposure to aged NMs will help us more closely approach realistic exposure conditions. Then, we will be able to decipher the transfer of CeO_2_ NMs at different stages of the lifecycle to various organs in animals, their permeation of biological membranes, accumulation in reproductive organs, and their impact on embryonic development, as conducted with pristine NMs [67,77,78,79,80]. To go further in the relevance of the exposure scenario, the complexity of the exhaust emissions [81] should be considered when assessing their genotoxicity. For instance, a considerable amount of Polycyclic Aromatic Hydrocarbons (PAHs) and their alkylated derivatives are emitted by diesel engines [82]. Some of them are well known for their carcinogenic and reprotoxic potential, such as benzo[a]pyrene (B*a*P) [83]. An even more relevant exposure scenario can assess the potential hazard due to co-exposure to combusted CeO_2_ NMs associated with other organic compounds generated during diesel combustion.

## Figures and Tables

**Table 1:** Biological variability of the data. Median values of % Tail DNA of each condition of three experiments, with 1st and 3rd quartiles.

Rat Sperm Cells	MEDIAN Values	1st Quartile	3rd Quartile	Human Sperm Cells	MEDIAN values	1st Quartile	3rd Quartile
Negative control	9.34	7.94	11.24	Negative control	10.8	7.26	13.7
1 µg·L^−1^ CeO_2_	18.2	17.17	23.47	1 µg·L^−1^ CeO_2_	30.1	28.28	32.06
10 µg·L^−1^ CeO_2_	14.46	10.48	17.72	10 µg·L^−1^ CeO_2_	26.24	21.69	27.09
100 µg·L^−1^ CeO_2_	11.31	10.64	15.13	100 µg·L^−1^ CeO_2_	21.74	13.72	23.99
1000 µg·L^−1^ CeO_2_	11.15	10.53	12.19	1000 µg·L^−1^ CeO_2_	14.91	12.88	17.02

## Supplementary Materials

The following are available online at https://www.mdpi.com/2079-4991/10/12/2327/s1. Figure S1: X-ray diffractogram and transmission electron microscopy image of the 850 °C aged CeO_2_ NMs.
