# Supplementary material for: CeO2 Nanomaterials from Diesel Engine Exhaust Induce DNA Damage and Oxidative Stress in Human and Rat Sperm In Vitro
Source: Nanomaterials (Basel). 2020 Nov 24;10(12):2327. doi: 10.3390/nano10122327 (PMC7760532; doi:10.3390/nano10122327)
Supplement: Supplementary file 1 [file nanomaterials-10-02327-s001.pdf]

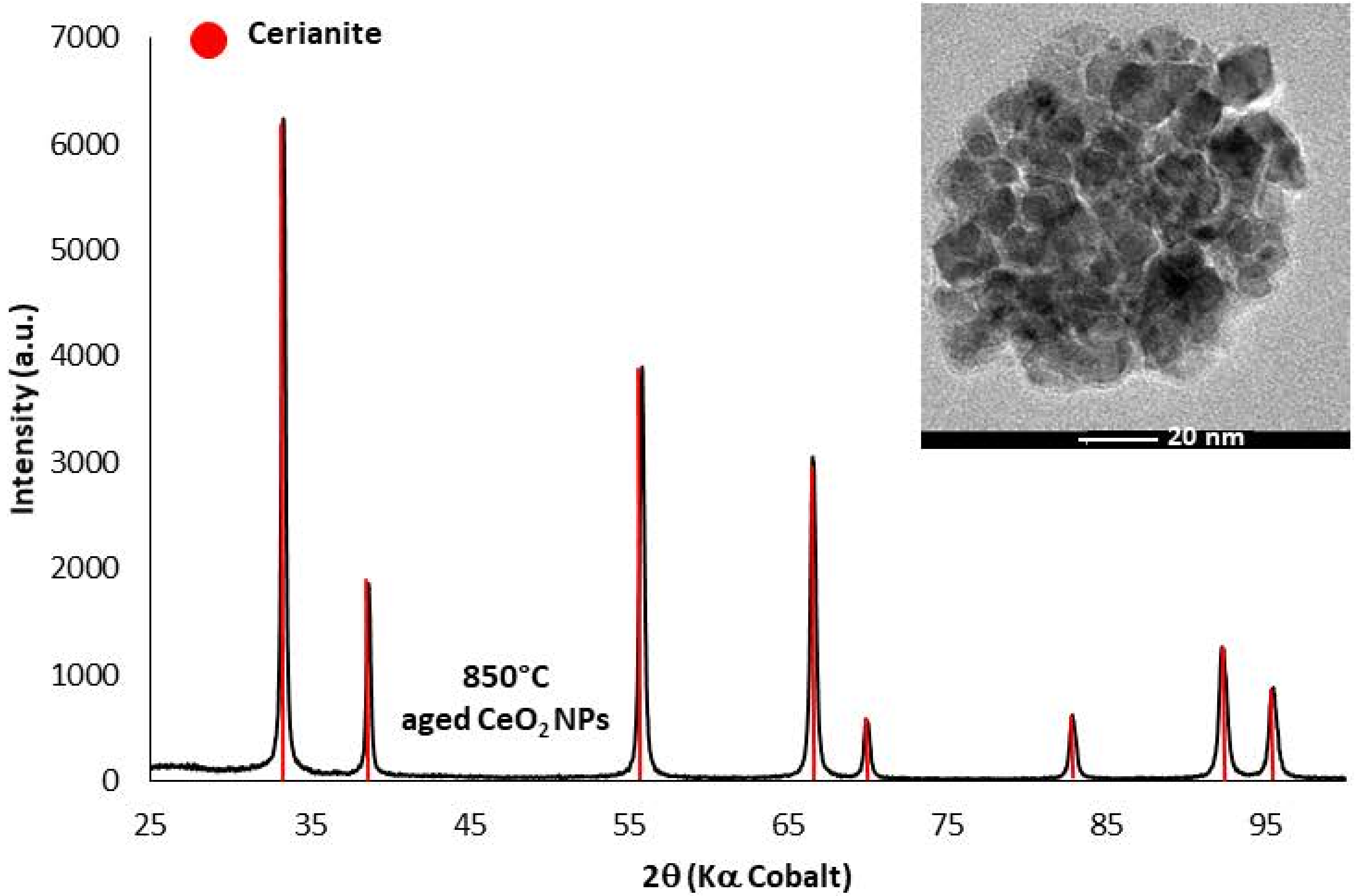

Figure SI1:X-ray diffractogram and transmission electron microscopy image of the 850°C aged CeO_2 NMs.
